# Green Synthesis and Characterization of Silver Nanoparticles Using a *Lythrum salicaria* Extract and In Vitro Exploration of Their Biological Activities

**DOI:** 10.3390/life12101643

**Published:** 2022-10-19

**Authors:** Andreia Corciovă, Cornelia Mircea, Ana Flavia Burlec, Adrian Fifere, Ioana Turin Moleavin, Alexandra Sarghi, Cristina Tuchiluș, Bianca Ivănescu, Irina Macovei

**Affiliations:** 1 Faculty of Pharmacy, “Grigore T. Popa” University of Medicine and Pharmacy, 16 University Street, 700115 Iasi, Romania; 2 Centre of Advanced Research in Bionanoconjugates and Biopolymers, “Petru Poni” Institute of Macromolecular Chemistry, 41A Grigore Ghica Voda Alley, 700487 Iasi, Romania; 3 Faculty of Medicine, “Grigore T. Popa” University of Medicine and Pharmacy, 16 University Street, 700115 Iasi, Romania

**Keywords:** Taguchi design, FTIR, EDX, antifungal, lipoxygenase, erythrocyte hemolysis

## Abstract

This research describes an eco-friendly green route for the synthesis of AgNPs using an aqueous extract of *Lythrum salicaria*. Taguchi design was used to optimize the synthesis method, taking into account various working conditions. The optimum parameters were established using a 3 mM AgNO_3_ concentration, a 1:9 extract:AgNO_3_ volume ratio, a pH value of 8, 60 ℃ temperature, and 180 min reaction time. The synthesized AgNPs were characterized using UV-Vis and FTIR spectroscopy, and TEM and EDX analysis. The SPR band at 410 nm, as well as the functional groups of biomolecules identified by FTIR and the EDX signals at ~3 keV, confirmed the synthesis of spherical AgNPs. The average AgNPs size was determined to be 40 nm, through TEM, and the zeta potential was −19.62 mV. The antimicrobial assay showed inhibition against *S. aureus* and *C. albicans*. Moreover, the results regarding the inhibition of lipoxygenase and of peroxyl radical-mediated hemolysis assays were promising and justify further antioxidant studies.

## 1. Introduction

Noble metal (e.g., Pt, Ag, Au) nanoparticles have gained researchers’ attention due to their multiple benefits in various fields, such as medicine, the food industry, material science, physics, and chemistry. In the biomedical field, noble metal nanoparticles are versatile agents used in drug delivery, diagnosis, radiotherapy enhancement, photo-ablation procedures, and hyperthermia studies [1,2]. The photophysical metal properties, such as facile synthesis in different shapes and sizes, easy derivatization with chemical and biomolecular ligands, biocompatibility, and high stability, represent the most appropriate attributes of nanoparticles, recommending them to industry [3]. Among such nanoparticles, silver nanoparticles (AgNPs) have gained special attention, due to their superior electrical conductivity, chemical stability, controlled geometry, catalytic and antibacterial properties [4]. AgNPs are used for many medical applications, including sensing devices, coating materials, catheters, bone cement and wound dressings [5].

Several methods can be applied for AgNPs synthesis, each presenting advantages and disadvantages. A general classification divides them into bottom-up and top-down techniques. In the top-down category, the principle relies upon reducing the size of bulk material by mechanical milling, laser ablation or sputtering. Although the final product presents uniform physico-chemical properties, these methods also involve high energy consumption. The bottom-up techniques use molecules or atoms to prepare nanoparticles through various procedures that can be either physical and chemical (vapor deposition, chemical reduction, spray/flame pyrolysis, electrodeposition, supercritical fluid precipitation, microwaves) or biological (using bacteria, fungi, yeast, algae, plant extracts). Nowadays, biological methods are of high interest as they allow the overcoming of several drawbacks, such as using unsafe organic solvents that may be dangerous to human life and the environment, complicated synthesis procedures with many intermediate steps, expensive equipment or reagents, and high energy consumption. Thus, synthesis using plant material presents the advantages of lack of pathogenicity and production of homogenous nanoparticles. Secondary metabolites in plant extracts, such as polyphenols, flavonoids, terpenoids and alkaloids, reduce the metallic precursor and stabilize the surface. The reaction between the plant extract and the silver salt proceeds under the influence of reaction conditions, such as silver ions concentration, temperature, pH and period of time for magnetic stirring [6]. Through this process, eco-friendly nanoparticles are generated [7]. Using a plant extract for synthesizing AgNPs designed for drug delivery also confers the advantages of easy access to the bloodstream and cells that accumulate at the target site, and high biocompatibility, allowing sustained drug release. Even so, the method has disadvantages, and the most discussed is the degree of toxicity, which is not fully understood and quantified [8,9].

*Lythrum salicaria* L. (purple loosestrife) is a native Eurasian flowering plant that is nowadays naturalized in most temperate areas of the world, being a member of the *Lythraceae* family [10]. This species has been known in folk medicine for its multiple biological activities for centuries. Internal administration of powdered herb and liquid forms (infusion, decoction, extract, syrup) may treat dysentery, chronic and acute diarrhea, chronic intestinal catarrh, hemorrhoids and eczema. It also presents external use in eye inflammation, sinusitis, varicose veins, bleeding gums and ulcerations [11]. The chemical composition of the aerial part of *L. salicaria* includes polyphenols, along with nonpolar constituents, such as steroids (β-sitosterol; daucosterol), triterpenes (oleanolic acid; corosolic acid; ursolic acid; betulinic acid), phthalates (isobutyl phthalate), coumarins (buntansin; peucedanin) and alkaloids (lythranine; lythranidine; lythramine). Polar phytochemicals in *L. salicaria* belong to the class of ellagitannins (vescalagin; castalagin; salicarinin A/B/C; pedunculagin; lythrine A/B/C/D), flavonoids (luteolin; orientin; apigenin; vitexin; rutin), anthocyanins (malvidin 3,5-di-*O*-glucoside; cyanidin 3-*O*-glucoside), and phenolic acids (ellagic acid; caffeic acid; chlorogenic acid; ferulic acid; gallic acid; syringic acid; vanillic acid). The rich composition in biologically active principles justifies the several promising activities shown in in vitro and in vivo studies. Oral administration of extracts from the aerial part/flowering top of *L. salicaria*, obtained using various solvents (water, ethanol, hexane, chloroform) tested on animal models, proved to have antidiarrheal and anti-inflammatory activities, along with a hypoglycemic effect [11,12]. Moreover, *L. salicaria* extracts showed remarkable antioxidant, antimicrobial and antitumoral activities [13,14,15].

Successful biological AgNPs synthesis using *L. salicaria* extract has recently been reported in the literature, although the studies were conducted in order to incorporate the nanoparticles in nanohybrids, with cellulose, chitosan and lignocellulose [16]. Other comparative research was conducted on AgNPs synthesized using aerial parts and root extracts [17]. Thus, the novelty of the present study is associated with AgNPs synthesis via a *L. salicaria* aqueous extract, establishing optimal reaction conditions, for the first time, using the Taguchi design. Applying the Taguchi model to optimize AgNPs synthesis is an advantage, since it is a fast and efficient method that provides information on different parameters and levels in the same experiment. Here, we characterized the synthesized AgNPs using physico-chemical attributes and microbiological and antioxidant activities. In order to achieve valuable physico-chemical data, UV-Vis Spectroscopy, Dynamic Light Scattering (DLS), Transmission Electron Microscopy (TEM), Energy Dispersive X-ray analysis (EDX) and Fourier Transform Infrared (FTIR) spectroscopy were used. Moreover, to the best of our knowledge, the present study is the only one that has used, for antioxidant activity testing, the determination of the inhibition of lipoxygenase and of peroxyl radical-mediated hemolysis for AgNPs obtained using *L. salicaria* aqueous extract.

## 2. Materials and Methods

### 2.1. Obtaining of Extracts

For the preparation of the *L. salicaria* extract, 10 g of plant material, as grounded aerial part (purchased from a local natural and biological products market), were added to 100 mL distilled water, at an adjusted temperature of 40 ℃, while continuously stirring at 500 rpm for 30 min. After the mixture was cooled at room temperature, filtration was carried out using a Whatman filter paper no. 1 and the filtrate was stored at 4 ℃ for further experiments.

### 2.2. Preparation and Optimization of Nanoparticles

The Taguchi design was used to study AgNPs synthesis conditions in an experiment that uses minimal resources and, in our case, tested five parameters. AgNPs were synthesized by adding different concentrations of extract to silver nitrate (AgNO_3_) solution, in various volume ratios. These mixtures were subjected to magnetic stirring at 500 rpm, at different adjusted pH values, using 1 M HCl and 1 M NaOH, at different temperatures and time intervals. The change in color was monitored and the maximum absorbance was recorded in the 300–600 nm range by UV-Vis spectroscopy. The analysis was performed in triplicate.

The formed AgNPs were separated by centrifugation at 8000 rpm for approximately 30 min. After removing the supernatant, the AgNPs were purified by washing three times with distilled water and eventually dried at 40 ℃ until constant mass.

The theoretical concentration of AgNPs in the colloidal synthesis mixture was calculated using a mathematical method [18,19]. Firstly, the average number of atoms per nanoparticle (N) was determined through Equation (1):

(1) $$N=\frac{\left(\pi \rho D^{3}N_{A}\right)}{6M}$$

where π = 3.14, *ρ* = 10.5 g/cm^3^ (density of face centered cubic silver), D = 133.5 × 10^−7^ cm (average diameter of nanoparticles), M = 107.868 g/mol (atomic mass of silver) and N_A_ = 6.023 × 10^23^ mol^−1^ (Avogadro’s number).

Secondly, the molar concentration of nanoparticles in the resulting colloidal mixture (*C*) could be calculated only by considering that all silver ions (Ag^+^) were entirely converted to AgNPs in the biosynthesis process. Equation (2) was applied for the determination of *C*:

(2) $$C=\frac{N_{T}}{\left(NVN_{A}\right)}\;$$

where *N_T_* = total amount of Ag atoms added as AgNO_3_ (3.0 mM = 0.003 M), *N* = average number of atoms per nanoparticle, *V* = reaction volume (0.22 L) and *N_A_* = Avogadro’s number [18,19].

### 2.3. AgNPs Characterization

Regarding the physico-chemical characterization of AgNPs, the change in mixture color was initially visually monitored and then the UV-Vis spectra were recorded, observing the presence of the peak due to surface plasmon resonance (SPR). The absorbances were recorded with a Jasco V-530 UV-Vis double beam spectrophotometer (Tokyo, Japan) in the 300–600 nm range. The functional groups involved in the green synthesis of AgNPs were analyzed by FTIR spectroscopy (Bruker Vertex 70 spectrophotometer, Bruker, Billerica, MA, USA) in the 4000–310 cm^−1^ range, comparing the extract and AgNPs’ spectra. The other characteristics of AgNPs were examined by the following: DLS (the hydrodynamic diameter and the zeta potential), using a Delsa Nano Submicron Particle Size Analyzer (Beckman Coulter Inc., Fullerton, CA, USA); TEM (dimensions and morphology), using a Hitachi High-Tech HT 7700 Transmission Electron Microscope (Hitachi High-Technologies Corporation, Tokyo, Japan; EDX (elemental composition), using a Quanta 200 Environmental Scanning Electron Microscope (ESEM) with EDX (FEI Company, Brno, Czech Republic).

Furthermore, the total phenolic content of the *L. salicaria* extract (diluted correspondingly) and supernatant (after the first separation of AgNPs) was performed using a previously described UV-Vis spectrophotometric method, with gallic acid as standard [20]. The analysis was performed in triplicate and the results were expressed as mg gallic acid equivalents (GAE) per mL sample.

### 2.4. Antimicrobial Testing

The synthetized AgNPs and the corresponding extract were investigated for antimicrobial activity, using disk diffusion methods [21,22], against different bacterial (*Staphylococcus aureus* ATCC 25923, *Pseudomonas aeruginosa* ATCC 27853) and fungal (*Candida albicans* ATCC 90028) pathogens, obtained from the Culture Collection of the Department of Microbiology, “Grigore T. Popa” University of Medicine and Pharmacy, Iasi, Romania. Standard culture mediums, such as Mueller–Hinton agar (Biolab) for fungi and Mueller–Hinton agar (Oxoid) for bacteria, were used. After the samples and positive controls (discs containing 5 µg ciprofloxacin and 25 µg fluconazole, respectively) were applied, the plates were incubated at 35 ℃ for 24 h. All experiments were conducted in triplicate, and, after the inhibition zones were measured (mm), the mean ± standard deviation was calculated. The Minimal Inhibitory Concentration (MIC) and Minimal Bactericidal Concentration (MBC) or Minimal Fungicidal Concentration (MFC), were determined by the broth microdilution method. Subsequent double dilutions of the tested samples in Mueller-Hinton broth were inoculated with the suspension of the microorganism test.

### 2.5. Antioxidant Activity

The antioxidant activities of the samples (AgNPs and extract) were determined using 15-lipoxygenase (LOX) inhibition and peroxyl radical-mediated hemolysis inhibition assays. Regarding the first method, after the mixture of lipoxygenase (in pH 9 borate buffer solution) and samples were left to stand in the dark at room temperature for 10 min, linoleic acid (in pH 9 borate buffer solution) was added, and the absorbances were recorded at 234 nm [23]. The second method focused on the determination of the inhibition capacity of erythrocyte hemolysis, mediated by peroxyl free radicals [24]. Samples treated with a solution of 2,2′-azobis-(2-amidinopropane) dihydrochloride (AAPH) (in phosphate buffer pH 7.4) and an erythrocyte suspension (10 % in 0.9 % saline) were maintained for 3 h at 37 ℃, cooled to room temperature, diluted with phosphate buffer (pH 7.4) and, eventually, centrifuged for 10 min. Simultaneously, a control solution containing only AAPH and erythrocyte suspension was prepared. The absorbances were recorded at 540 nm. All experiments were performed in triplicate and gallic acid was used as standard. For samples that showed an activity of more than 50 %, the EC_50_ value was also calculated and expressed as µg extract/mL final solution.

## 3. Results

### 3.1. Taguchi Design Experiment for Optimization of Reaction Parameters

The optimization of parameters for more efficient AgNPs synthesis was based on an L9 orthogonal array design, a method that combines mathematical and statistical principles in order to obtain a predictive knowledge of a complex process with several variables, but with a reduced number of attempts [25,26]. This design presents several advantages over the traditional more laborious and time-consuming optimization methods. In the traditional methods each variable is evaluated one by one, while the others remain constant, and, therefore, data on what happens when variables change simultaneously is not obtained [27].

The model was obtained using variables presented in Table 1 at levels 1, 2 and 3.

In order to process the data, the *S/N ratio* was calculated using Equation (3). The *S/N ratio* measures the quality characteristic deviating from the desired value, and represents the ratio of the target value (signal) to the standard deviation for the response variable (noise), which can be calculated by selecting the formula corresponding to the quality characteristic “larger is better”.

(3) $$S/N\;ratio\;\left[dB\right]=-10\;log\left[(1/y_{1}{}^{2}+1/y_{2}{}^{2}+1/y_{3}{}^{2}+\ldots +1/y_{n}{}^{2}\right)/n]$$

where: *n* = number of experiments, *yi* = response variable for the experiment (absorbance) [26,27].

The obtained results for the *S/N ratio* are presented in Table 2. Each line in the matrix contains the numbers corresponding to the level at which the factors A, B, C, D and E are expressed in Table 1.

**Figure 1:**
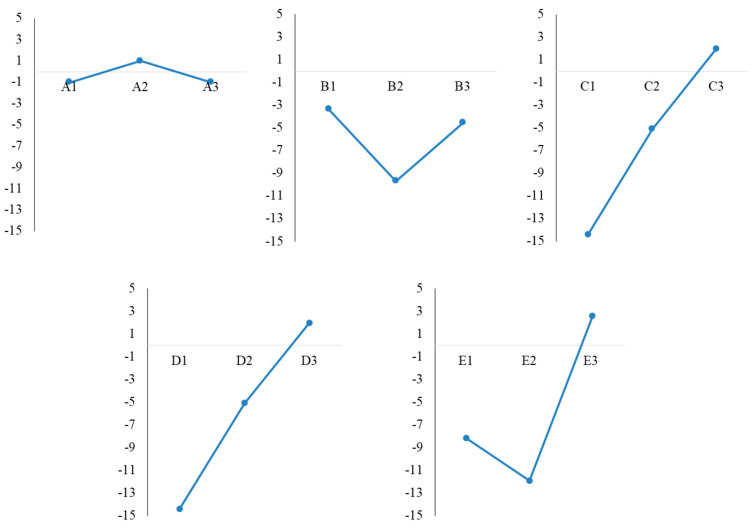
Taguchi design—main effects plot of mean of *S/N ratio* for AgNPs synthesis.

Taking into account the average value of absorbance and the *S/N ratio* calculated for each level, the optimal synthesis conditions were A3B1C3D3E3 (line L7, A at level 3, B at level 1, C, D and E at level 3), followed by A2B1C2D2E3 (L4), and, therefore, we could say that, in both cases, a volume ratio of 1:9 extract:AgNO_3_ and 180 min reaction time were optimal. The average *S/N ratio* for each level is presented in Table 3 and the main effect of each variable is graphically represented in Figure 1. In order to establish the optimal synthesis conditions, the maximum S/N value was investigated.

Considering the *S/N ratio*, the optimum conditions for AgNPs synthesis were A2B1C3D3E3, with a 3 mM AgNO_3_ concentration, 1:9 extract:AgNO_3_ ratio, a pH value of 8, 60 ℃ temperature and 180 min reaction time.

To the best of our knowledge, the present study is the only one that has used the Taguchi model to establish the reaction conditions for AgNPs synthesized using an *L. salicaria* extract. Moreover, the traditional optimization of synthesis can be found in a single study carried out by Srećković et al., which led to the following conditions: 20 mM AgNO_3_ concentration, 25 ℃, pH 12, and 30 min reaction time [17]. In both cases, the synthesis was optimal at alkaline pH, which can be explained by the change in dissociation constants values for functional groups involved in the reduction process, which leads to an increase in the availability of compounds for the synthesis process [28].

### 3.2. Physico-Chemical Characterization of AgNPs

AgNPs synthesis by means of green methods represents the focus of scientists, given that they imply lower costs and ease in the monitoring and sampling processes. Moreover, plants are easy to grow and safe to handle, and, implicitly, this type of eco-friendly synthesis could replace chemical techniques for obtaining AgNPs [29]. The steps implied by AgNPs synthesis are represented by the reduction of Ag^+^ and agglomeration of colloidal nanoparticles with oligomeric clusters formation [30].

**Figure 2:**
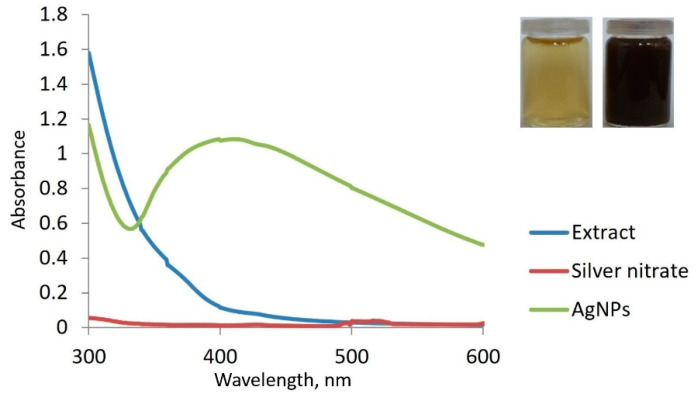
Comparative spectra of extract, AgNO_3_ and AgNPs; Inset: visual observation of color change in time.

The first indicator of AgNPs synthesis is noticed through visual observation, but the formation of nanoparticles must be confirmed by other methods as well. Therefore, we continued monitoring the color change by means of UV-Vis spectroscopy (Figure 2).

As seen in the inset of Figure 2, the initial color of the extract was yellow, changing to dark brown after adding AgNO_3_ under specific conditions. When analyzing the UV-Vis spectra, no absorption peak was observed in the 300–600 range for either the extract or AgNO_3_ solution, but a distinct peak at 410 nm was revealed for the obtained colloidal solution. This peak could be due to excitation of SPR [31], which could determine the optical, physical and chemical properties of AgNPs [29]. The presence of a single SPR peak could explain the spherical shape of AgNPs [32]. The obtained results were in accordance with other studies focusing on AgNPs synthesized using *L. salicaria* extracts, in which the SPR band was observed at 415 nm [16] or in the 396–415 nm range [17]. The calculated concentration of AgNPs in the colloidal solution was found to be 2 × 10^−10^ mol/L.

Differences in the position and appearance of the peak, even for the same plant species, can be explained by different harvesting areas, different conditions for extract preparation and for AgNPs synthesis. Nonetheless, these conditions can also lead to modifications in the shape and size of AgNPs. A wide peak generally indicates larger particles [29]. Moreover, their shape can be predicted considering the position of the SPR band, for example, in the 400–490 nm range, the particles are spherical [33].

The mechanism of AgNPs synthesis is explained through biomolecules present in the extract (polyphenols, phenolic acids, phytosterols, alkaloids, proteins, enzymes, sugars, etc.), which are compounds capable of donating electrons, so the reduction process from Ag^+^ to Ag^0^ can take place. This process is generally followed by agglomeration of free silver atoms and, eventually, by the formation of the AgNPs colloidal solution. Such biomolecules also participate in the functionalization and stabilization of AgNPs [34,35]. For example, there are many studies that demonstrate that phenolic acids are some of the most important bioactive substances that participate in AgNPs synthesis and even more in the process of stabilization of nanoparticles, therefore, having a synergistic effect. Yee-Shing Liu et al. [36] combined their research with that of other studies and proposed a mechanism for AgNPs formation using caffeic acid, one of the phenolic acids present in rice husk extract, along with gallic, protocatechuic, ferulic, vanillic and syringic acids. Caffeic acid in alkaline medium releases electrons, that are transferred to Ag^+^, which is reduced to Ag^0^. On the other hand, caffeic acid is transformed into a free radical which, in turn, reduces another Ag^+^ and is converted to ortho-quinone. By the coupling of two free radicals of caffeic acid, an oxidative dimer is formed which releases 4 electrons that participate in the reduction of Ag^+^, being transformed into quinone. The formed quinones can bind to AgNPs and cause steric hindrance, thus, stabilizing the particles [36]. Flavonoids can also donate electrons or hydrogen and the keto form of the nucleus reduces Ag^+^ to Ag^0^ [33]. Thus, the total phenolic content of the extract used for synthesis (in suitable dilution) and of the first collected supernatant, were measured, so as to estimate the amount of such compounds involved in the synthesis process. If, initially, the phenolic content of the extract was 1.2404 mg GAE/mL, after the first separation, the supernatant only had a remaining content of 0.1122 mg GAE/mL, which could confirm the participation of bioactive compounds in the synthesis process.

Other examples of biosubstances involved in AgNPs synthesis are triterpenes. Aazam et al. [37] proposed a mechanism for the synthesis of AgNPs using ursolic acid, the main constituent of an *Ocimum sanctum* extract. Following the redox reaction, the -OH group of the ursolic acid structure deprotonates and oxidizes with the formation of a radical, promoting the reduction of silver from Ag^+^ to Ag^0^. Further agglomeration and formation of oligomeric clusters occurs. During these steps, several species of AgNPs can be formed, given that Ag^+^ reacts with Ag^0^, forming Ag_2_^+^, which dimerizes to Ag_4_^+2^.

**Figure 3:**
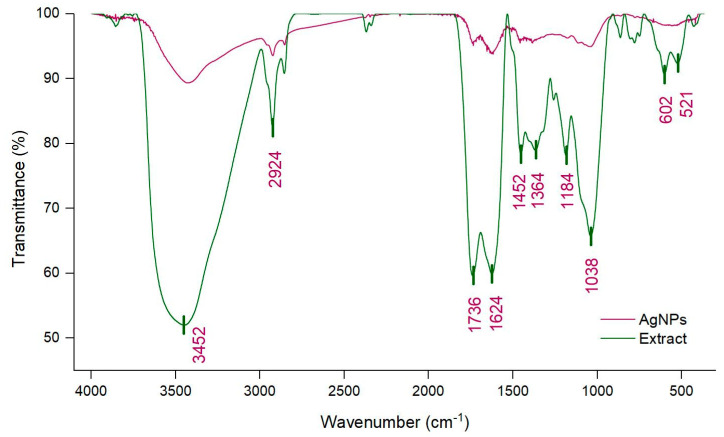
FTIR spectra of *L. salicaria* extract and of synthesized AgNPs.

In order to highlight the functional groups and, implicitly, the compounds that participate in AgNPs synthesis, FTIR analysis was used. Figure 3 presents the comparative FTIR spectra of the extract and of the corresponding AgNPs.

The FTIR spectrum of the extract shows a broad absorption peak at 3452 cm^−1^, which was related to the stretching vibration of -OH groups found in alcohols and phenols and to the N-H stretching from amides. The peaks at 2924 cm^−1^ and 2854 cm^−1^ corresponded to C-H stretching and bending vibrations of CH_3_ and CH_2_ (alkanes) [19,28]. The bands detected at 1736 cm^−1^ and 1624 cm^−1^ were connected to the vibration of C=O from amides and carboxylic groups, and -C=C- aromatic rings, respectively [38]. The bands at 1452 cm^−1^ and 1364 cm^−1^ could be attributed to stretching vibrations of CO of carboxylic acids or esters, to bending vibration of N-H in amides or to stretching vibrations of N-H in secondary amines, or to the C-N stretch vibration of aromatic amines. Furthermore, the 1184 cm^−1^ and 1038 cm^−1^ bands could be attributed to the C-O stretching vibrations of alcohols, esters, ethers and carboxylic acids from terpenoids and flavonoids and to the C-O-C stretching of aromatic ethers and polysaccharides [39,40]. The peaks found in the 600–900 cm^−1^ range corresponded to CH out of plane bending vibrations and the peak at 524 cm^−1^ could be responsible for C=C torsion and ring torsion of phenyl, or to C-N stretch from secondary amines and amides [40,41]. Generally, similar peaks, but also shifts, reduction or disappearance of peaks, from the extract spectrum could be observed in the FTIR spectra of AgNPs. The disappearance of peaks could be explained by the participation of these groups only in the reduction process, while modifications in peak position and intensity could indicate the involvement of the corresponding groups not only in the reduction, but also in the stabilization processes [39]. Therefore, biomolecules, such as phenolic acids, flavonoids, terpenoids and proteins, could participate in the reduction and stabilization of AgNPs. The obtained results were comparable to those obtained by Samira Mohammadalinejhad et al. and by Srećković et al. [16,17]. The first of the aforementioned groups of researchers suggested that polyphenols are involved in the reduction process, while flavonoids, polyphenols, tannins and gallic acid are responsible for the reduction process, as well as for the stabilization of nanoparticles.

**Figure 4:**
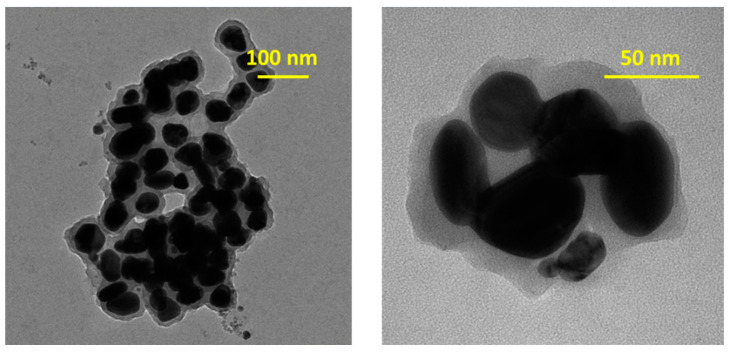
TEM image of the synthesized AgNPs.

**Figure 5:**
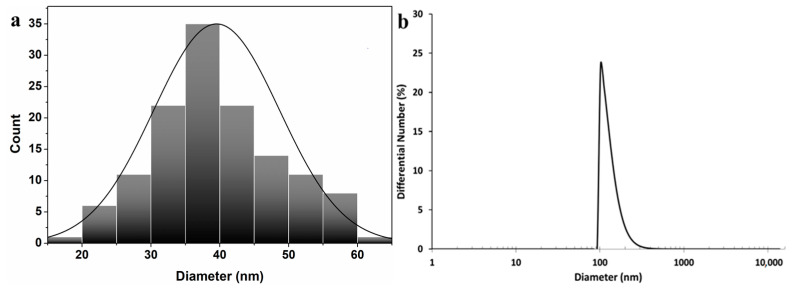
TEM histogram curve fitting diameters (**a**) and DLS size distribution (**b**).

The morphological analysis of AgNPs was examined by TEM (Figure 4) and the corresponding histogram can be found in Figure 5a.

Figure 4 exhibits a good distribution of spherical AgNPs, with a relative uniformity in size, in the 40 nm range. The presence of a grey cloud region around AgNPs can be explained by the presence of organic compounds on the surface of nanoparticles, with a role in stabilizing their surface, thus, preventing possible agglomeration [36]. The negative zeta potential (the charge around a moving particle in the colloidal solution in electric field) of −19.62 mV could have been due to biomolecules found on the AgNPs surfaces, that might determine a repulsion between particles and implicitly prevent agglomeration [33]. This value was in accordance with that obtained by Samira Mohammadalinejhad et al. [16], namely −20 mV.

The DLS analysis revealed an average AgNPs hydrodynamic dimension of 133.5 nm (Figure 5b). Other authors obtained values in the 20–138 nm range [17] or an average of 50 nm [16]. The TEM analysis measured only the average diameter of the metallic silver core so it did not include any coating/stabilizing agent. On the other hand, the DLS analysis measured the dynamic fluctuation and velocity of particles in suspended clusters, meaning the metallic silver core and the molecules that were attached to the surface nanoparticles, which could, depending on the structure, undergo a process of solvation and expansion in solution. Therefore, the hydrodynamic diameter estimated by DLS was larger than the size estimated by TEM [33,42,43].

**Figure 6:**
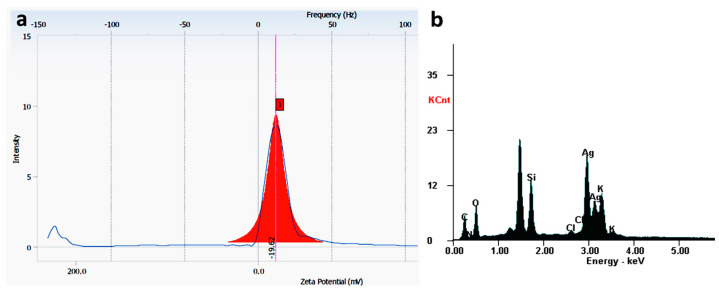
Zeta potential (**a**) and EDX spectra (**b**) of AgNPs.

The quantitative elemental structure of AgNPs was investigated by EDX analysis (Figure 6).

Data analysis revealed that EDX spectra of AgNPs mainly contained a specific and intense peak at 3 keV for Ag (37.52 wt %), but also C (18.83 wt %), O (24.49 wt %) and small quantities of N, Br, Si, Cl and K. Consequently, the results confirmed the synthesis of AgNPs. The presence of other elements might be related to the breakdown of capping agents from the surface of nanoparticles.

### 3.3. Antimicrobial Activity

Firstly, the disk diffusion method was used, which is a routine, simple and low-cost antimicrobial susceptibility test. The antimicrobial activity was assessed using two bacterial strains, *Staphylococcus aureus* (*S. aureus*) and *Pseudomonas aeruginosa* (*P. aeruginosa*) and a fungal strain, *Candida albicans* (*C. albicans*). The results are presented in Table 4.

The AgNPs presented better activity, compared to the extract against the Gram-positive bacteria and pathogenic yeast. However, no activity was detected for either in the case of the Gram-negative bacteria.

Further, the broth microdilution method was applied, in order to determine the MIC and MBC values, and since it represents a standardized method, the results could be of clinical relevance [44]. Implicitly, the MIC and MBC/MFC values of samples against *S. aureus* ATCC 25923 and *C. albicans* ATCC 90028 were determined and are presented in Table 5.

MIC was the lowest concentration of the sample at which bacterial growth was completely inhibited after 24 h incubation at 37 °C. In the study carried out by Srećković et al. [17], the MIC value obtained for AgNPs was 0.31 mg/mL for *S. aureus* and 0.62 for *C. albicans*. In our study, we obtained the same value for *S. aureus*, but for *C. albicans* the value was smaller (0.03 mg/mL).

To the best of our knowledge, the MBC value for AgNPs synthesized from *L. salicaria* has not been reported until now. The highest dilution showing 100 % inhibition was 0.62 mg/mL for *S. aureus* and 0.31 for *C. albicans*. For both tested microorganisms, the obtained results for MIC and MBC/MFC for the synthesized AgNPs were better than those obtained for the extract.

Indeed, most literature studies show better antibacterial activity of AgNPs on Gram-negative bacteria than on Gram-positive bacteria [45]. However, there are several research works that prove the contrary, as in our case. For example, Yage Xing et al. obtained better antibacterial activity on *S. aureus*, compared to *E. coli* for AgNPs formed using mango peel [46]. A similar example is that of AgNPs synthetized using olive leaves extract [47]. An explanation for the higher susceptibility in the case of Gram-positive bacteria, compared to Gram-negative bacteria, could be related to the differences between the wall structures of the two types of bacteria. The cell wall of Gram-positive bacteria consists of a thick peptidoglycan layer with teichoic acid and lipoteichoic acid, while the Gram-negative bacterial cell wall is more complex, containing an extra outer lipid membrane, with lipopolysaccharides, which could make the entrance of hydrophobic substances more difficult [48]. Therefore, a reason for better antimicrobial action in the case of Gram-positive bacteria could be represented by the more facile interaction between AgNPs and bacteria [49].

Moreover, the antimicrobial activity depends on the size and shape of AgNPs. Nanoparticles of smaller sizes, with a diameter of approximately 1–10 nm, have a higher surface:volume ratio and more efficient antimicrobial activity, interacting preferentially with bacteria [50]. Truncated triangular AgNPs have the strongest biocidal action compared to those that are spherical or rod shaped [51].

The mechanism surrounding the antibacterial activity has not been fully elucidated, but several explanations are possible, taking into account that AgNPs can adhere to, or can pass through, cell walls/membranes of microorganisms, or induce cellular toxicity and ROS generation, or modulate cell signaling.

The first proposed mechanism is that, following the electrostatic attraction between AgNPs and the cell membrane, nanoparticles tend to adhere to the membrane, which leads to alteration of the structure and rupture of the cell wall. Moreover, the interaction between AgNPs and sulfur-containing proteins found in the cell wall can lead to a chain reaction, starting with structural modifications, affecting the transport process and increasing permeability, which leads to cell content (ions, proteins, reducing sugars) leakage and, sometimes, ATP synthesis inhibition.

The second mechanism consists of the penetration of AgNPs inside the cell and nucleus, which can modify cellular functioning by interacting with the cell structure or biomolecules. Therefore, the destabilization and denaturation of proteins containing thiol groups can occur via interaction with silver ions, or AgNPs, and silver ions can interact with disulfide bonds, thus, blocking the active binding site, leading to functional deficiencies in microorganisms. Moreover, AgNPs can interact with DNA, and through the reaction of Ag^+^ ions with nucleic acids, can lead to the destruction of the double helix structure.

For the third mechanism, a high concentration of Ag^+^ ions produce cellular oxidative stress by generation of radicals and ROS. Free radicals can cause destruction of the mitochondrial membrane and can interact with lipids, enhancing lipid peroxidation. ROS generation can lead to hyperoxidation of lipids, proteins and DNA.

The last possible mechanism focuses on the modulation of the cellular signal system, which can lead to modifications in bacterial growth, as well as affect the processes at molecular and cellular levels [52,53].

### 3.4. Antioxidant Activity

Given that Srećković et al. [17] determined the antioxidant potential of an *L. salicaria* extract, and of the corresponding AgNPs, by DPPH and ABTS scavenging activity methods, and the results showed for the extract from the aerial parts slightly better activity (86.38 ± 0.13 µg/mL by DPPH method and 65.33 ± 2.08 µg/mL by ABTS test) versus AgNPs (>100 µg/mL by DPPH and 141.66 ± 17.05 µg/mL by ABTS test) [17], we proposed the use of other methods, with different principles. Therefore, our study focused on the determination of lipoxygenase inhibition and of peroxyl radical-mediated hemolysis inhibition capacities, in the 0.1562–5 mg/mL range. The results are presented in Table 6 and Table 7.

The inhibition of lipoxygenase, which was determined using the modified Malterud method [23], can be explained by polyphenolic compounds present in the extract that have the ability to block the activity of lipoxygenase, which catalyzes the oxidation of linoleic acid, thus, reducing the absorbance measured at 234 nm. The inhibition activity was calculated using the following formula:

(4) $$Activity\;\left(\%\right)=\left(A_{E}-A_{EI}\right)\times 100/A_{E}$$

where *A_E_* is the difference between the absorbances of the enzyme solution without inhibitor at 90 and 30 s and *A_EI_* is the difference between the absorbances of the enzyme-inhibitor solution at 90 and 30 s, respectively.

Lipoxygenases (5-, 12-, 15-lipoxygenase) are metalloenzymes that contain ferrous or ferric ions in their catalytic center, depending on the stage of the redox reaction (oxidation or reduction) [54]. Lipoxygenases catalyze the oxidation of unsaturated fatty acids with the formation of lipid peroxides, which can cause the spread of oxidation reactions or cause the oxidation of lipids, proteins, nucleic acids, thereby affecting their biological properties [55]. Uncontrolled activation of these enzymes causes oxidative stress and inflammation, and can lead to neurodegenerative diseases, atherosclerosis, diabetes or cancer [56]. AgNPs show a more intense antioxidant activity compared to the extract, the EC_50_ value being also slightly better than that of gallic acid. Their influence on lipoxygenase is most probably achieved through changes in the spatial structure of the enzyme or of its active center [56].

Antioxidant compounds can also block the peroxyl radical synthesis induced by AAPH, with consequent protection of the erythrocyte membrane. The reduction in the concentration of peroxyl radicals determines the decrease of the absorbance measured at 540 nm [24]. The erythrocyte hemolysis inhibition was calculated using the formula:

(5) $$Activity\;\left(\%\right)=100\times \left(A_{AAPH}-A_{S}\right)/A_{AAPH}$$

where *A_S_* is the absorbance of the sample and *A_AAPH_* is the absorbance of the positive control.

AAPH is a prooxidant compound that causes the oxidation of hemoglobin to methemoglobin, consequently inducing hemolysis, and possibly affecting the lipophilic structure of the erythrocyte membrane [57]. The synthesized AgNPs were more active compared to the extract. However, both the extract and AgNPs can block the prooxidant and hemolytic action of AAPH, without causing hemolysis in its absence. Blocking AAPH activity is done mainly by compounds that have both hydrophilic groups, that are capable of interacting with AAPH, but also lipophilic structures that allow passage through the erythrocyte membrane and block the intracellular action of AAPH [57]. Such compounds also have a protective effect on subcellular structures, proteins and DNA and can, thus, block pathological phenomena caused by oxidative stress [58]. Generally, the prooxidant action of peroxyl radicals generated by AAPH is blocked by compounds with hydroxyl groups that are capable of neutralizing and stabilizing the formed radicals [59].

## 4. Conclusions

In the present study, AgNPs were obtained via a simple and eco-friendly method, using a robust design, namely the Taguchi method, in order to identify the optimal synthesis parameters, which was achieved, for the first time, for such nanoparticles. AgNPs were successfully synthesized using AgNO_3_ as a precursor and an *L. salicaria* extract as a reducing and capping agent, as demonstrated by FTIR analysis, which also revealed the functional groups found in the extract that are responsible for the obtaining of AgNPs. Moreover, the synthesis was confirmed through the presence of the SPR band, by both visual observation of color change and UV-Vis spectroscopy. The presence of silver was highlighted by EDX analysis, and the negative zeta potential indicated a stable AgNPs colloidal solution. The formed nanoparticles showed antimicrobial activity against *S. aureus* and *C. albicans*. The novelty of the research consisted of, besides establishing the optimal reaction conditions using the Taguchi design, testing the antioxidant activity through inhibition of lipoxygenase and peroxyl radical-mediated hemolysis, which showed promising results for the formed nanoparticles, as well as for MBC testing. Therefore, further studies are justified, with the synthesized AgNPs being potential resources for nanotechnological applications.

## Figures and Tables

**Table 1:** The tested parameters and the levels set according to the chosen design.

Factor	Levels		
	1st	2nd	3rd
A AgNO_3_ concentration M	1	3	5
B Extract:AgNO_3_ ratio	1:9	5:5	9:1
C pH	3	5	8
D Reaction temperature (℃)	20	40	60
E Reaction time (minutes)	60	120	180

**Table 2:** Taguchi L9 orthogonal array design.

Level	Factors					Average of Absorbance	*S/N Ratio*
	A	B	C	D	E		
L1	1	1	1	1	1	0.1165	−18.67
L2	1	2	2	2	2	0.2727	−11.28
L3	1	3	3	3	3	0.9084	−0.83
L4	2	1	2	2	3	1.5108	3.58
L5	2	2	3	3	1	1.2177	1.71
L6	2	3	1	1	2	0.5525	−5.15
L7	3	1	3	3	3	1.7779	4.99
L8	3	2	1	1	2	0.1066	−19.44
L9	3	3	2	2	1	0.4146	−7.64

**Table 3:** S/N response values.

	*S/N Ratio* Average		
	Level 1	Level 2	Level 3
A	−10.26	0.04	−7.36
B	−3.36	−9.67	−4.54
C	−14.42	−5.11	1.95
D	−14.42	−5.11	1.95
E	−8.20	−11.95	2.58

**Table 4:** Antimicrobial activity of the extract and of the corresponding AgNPs.

Sample/Standard	Diameter of Inhibition Zones (mm) ± SD		
	*S. aureus* ATCC 25923	*P. aeruginosa* ATCC 27853	*C. albicans* ATCC 90028
Extract	12.00 ± 0.00	NA *	19.06 ± 0.05
AgNPs	14.00 ± 0.00	NA *	21.00 ± 0.00
Ciprofloxacin	30.00 ± 0.00	30.33 ± 0.57	NT **
Fluconazole	NT **	NT **	29.00 ± 0.00

* NA—no activity detected; ** NT—not tested.

**Table 5:** MIC and MBC/MFC values of the extract and of the corresponding AgNPs.

Samples	*S. aureus* ATCC 25923		*C. albicans* ATCC 90028	
	MIC (mg/mL)	MBC (mg/mL)	MIC (mg/mL)	MFC (mg/mL)
Extract	0.62	1.25	0.15	1.25
AgNPs	0.31	0.62	0.03	0.31

**Table 6:** 15-LOX inhibition capacity (%) and EC_50_ values of the extract and AgNPs.

**Sample**	**0.1562 mg/mL**	**0.3125 mg/mL**	**0.625 mg/mL**	**1.25 mg/mL**	**2.5 mg/mL**	**5 mg/mL**	**EC_50_ (µg/mL Final Solution)**
Extract	8.87 ± 0.55	12.22 ± 0.53	18.88 ± 0.30	29.38 ± 1.04	45.43 ± 1.38	72.30 ± 1.75	46.90 ± 1.74 ^a^
AgNPs	31.07 ± 0.58	41.29 ± 0.69	51.48 ± 0.44	63.93 ± 1.66	74.55 ± 1.36	98.09 ± 0.20	9.40 ± 0.30 ^b^
Gallic acid	17.23 ± 1.69	28.15 ± 2.32	47.23 ± 1.58	61.15 ± 1.80	72.14 ± 2.81	89.25 ± 1.74	11.98 ± 0.97 ^a^

^a^*p* < 0.0001 (extremely statistically significant) extract vs. gallic acid; ^b^ *p* > 0.01 (statistically significant) AgNPs vs. gallic acid.

**Table 7:** Inhibition of erythrocyte hemolysis mediated by peroxyl free radicals and EC_50_ values of the extract and AgNPs.

Sample	0.1562 mg/mL	0.3125 mg/mL	0.625 mg/mL	1.25 mg/mL	2.5 mg/mL	5 mg/mL	EC_50_ (µg/mL Final Solution)
Extract	4.09 ± 0.18	7.92 ± 0.39	14.01 ± 0.56	21.67 ± 0.84	30.56 ± 1.04	38.26 ± 0.95	-
AgNPs	11.05 ± 0.31	16.58 ± 0.46	23.59 ± 0.63	35.51 ± 0.42	46.98 ± 0.97	57.45 ± 0.89	213.94 ± 13.4 ^a^
Gallic acid	26.48 ± 0.21	37.19 ± 0.14	49.63 ± 0.17	60.04 ± 0.01	73.51 ± 0.06	82.01 ± 0.01	44.83 ± 0.49

^a^*p* < 0.0001 (extremely statistically significant) AgNPs vs. gallic acid.

## Abbreviation

**Abbreviation**	**Meaning**
AAPH	2,2′-azobis-(2-amidinopropane) dihydrochloride
ABTS	2,2′-azino-bis(3-ethylbenzothiazoline-6-sulfonic acid
AgNPs	Silver nanoparticles
AgNO_3_	Silver nitrate
FTIR	Fourier transform infrared spectroscopy
DLS	Dynamic light scattering
DPPH	2,2-diphenyl-1-picrylhydrazyl
EDX	Energy dispersive X-ray analysis
EC_50_	Half maximal effective concentration
GAE	Gallic acid equivalents
LOX	15-lipoxygenase assay
MIC	Minimum inhibitory concentration
MBC	Minimum bactericidal concentration
N_A_	Avogadro’s number
ROS	Reactive oxygen species
ESEM	Environmental scanning electron microscope
SPR	Surface plasmon resonance
TEM	Transmission electron microscopy
UV-Vis	Ultraviolet-visible

## References

[1] Conde J., Doria G., Baptista P. (2012). Noble metal nanoparticles applications in cancer. J. Drug Deliv..

[2] Behzad F., Naghib S.M., Kouhbanani M.A.J., Tabatabaei S.N., Zare Y., Rhee K.Y. (2021). An overview of the plant-mediated green synthesis of noble metal nanoparticles for antibacterial applications. J. Ind. Eng. Chem..

[3] Jain P.K., Huang X., El-Sayed I.H., El-Sayed M.A. (2007). Review of some interesting surface plasmon resonance-enhanced properties of noble metal nanoparticles and their applications to biosystems. Plasmonics.

[4] Kumar V.P.P.N., Pammi S.V.N., Kollu P., Satyanarayana K.V.V., Shameem U. (2014). Green synthesis and characterization of silver nanoparticles using *Boerhaavia diffusa* plant extract and their anti bacterial activity. Ind. Crops Prod..

[5] Al-Radadi N.S. (2018). Artichoke (*Cynara scolymus* L.) mediated rapid analysis of silver nanoparticles and their utilisation on the cancer cell treatments. J. Comput. Theor. Nanosci..

[6] Marinescu L., Ficai D., Ficai A., Oprea O., Nicoara A.I., Vasile B.S., Boanta L., Marin A., Andronescu E., Holban A.-M. (2022). Comparative antimicrobial activity of silver nanoparticles obtained by wet chemical reduction and solvothermal methods. Int. J. Mol. Sci..

[7] Jamkhande P.G., Ghule N.W., Bamer A.H., Kalaskar M.G. (2019). Metal nanoparticles synthesis: An overview on methods of preparation, advantages and disadvantages, and applications. J. Drug Deliv. Sci. Technol..

[8] Begum S.J.P., Pratibha S., Rawat J.M., Venugopal D., Sahu P., Gowda A., Qureshi K.A., Jaremko M. (2022). Recent advances in green synthesis, characterization, and applications of bioactive metallic nanoparticles. Pharmaceuticals.

[9] Elfaky M.A., Sirwi A., Ismail S.H., Awad H.H., Gad S.S. (2022). Hepatoprotective effect of silver nanoparticles at two different particle sizes: Comparative study with and without silymarin. Curr. Issues Mol. Biol..

[10] Tunalier Z., Koşar M., Küpeli E., Çaliş İ., Başer K.H.C. (2007). Antioxidant, anti-inflammatory, anti-nociceptive activities and composition of *Lythrum salicaria* L. extracts. J. Ethnopharmacol..

[11] Piwowarski J.P., Granica S., Kiss A.K. (2015). *Lythrum salicaria* L.—Underestimated medicinal plant from European traditional medicine. A review. J. Ethnopharmacol..

[12] Bencsik T. (2014). Comparative Histological, Phytochemical, Microbiological, and Pharmacological Characterization of Some *Lythrum salicaria* L. Populations.

[13] López V., Akerreta S., Casanova E., García-Mina J., Cavero R., Calvo M. (2008). Screening of Spanish medicinal plants for antioxidant and antifungal activities. Pharm. Biol..

[14] Becker H., Scher J.M., Speakman J.-B., Zapp J. (2005). Bioactivity guided isolation of antimicrobial compounds from *Lythrum salicaria*. Fitoterapia.

[15] Khanavi M., Moshteh M., Manayi A., Reza Shams M., Vazirian M., Ajani Y., Nasser Ost S. (2011). Cytotoxic activity of *Lythrum salicaria* L.. Res. J. Biol. Sci..

[16] Mohammadalinejhad S., Almasi H., Esmaiili M. (2019). Simultaneous green synthesis and in-situ impregnation of silver nanoparticles into organic nanofibers by *Lythrum salicaria* extract: Morphological, thermal, antimicrobial and release properties. Mater. Sci. Eng. C.

[17] Srećković N.Z., Nedić Z.P., Liberti D., Monti D.M., Mihailović N.R., Katanić Stanković J.S., Dimitrijević S., Mihailović V.B. (2021). Application potential of biogenically synthesized silver nanoparticles using *Lythrum salicaria* L. extracts as pharmaceuticals and catalysts for organic pollutant degradation. RSC Adv..

[18] Alshehri A.A., Malik M.A. (2020). Phytomediated photo-induced green synthesis of silver nanoparticles using *Matricaria chamomilla* L. and its catalytic activity against rhodamine B. Biomolecules.

[19] Macovei I., Luca S.V., Skalicka-Woźniak K., Sacarescu L., Pascariu P., Ghilan A., Doroftei F., Ursu E.-L., Rimbu C.M., Horhogea C.E. (2022). Phyto-functionalized silver nanoparticles derived from conifer bark extracts and evaluation of their antimicrobial and cytogenotoxic effects. Molecules.

[20] Gird C., Nencu I., Popescu M., Costea T., Duţu L., Balaci T., Olaru O. (2017). Chemical, antioxidant and toxicity evaluation of rosemary leaves and its dry extract. Farmacia.

[21] CLSI (2020). Performance Standards for Antimicrobial Susceptibility Testing.

[22] Clinical and Laboratory Standards Institute (2009). M44-A2: Method for Antifungal Disk Diffusion Susceptibility Testing of Yeasts.

[23] Malterud K.E., Rydland K.M. (2000). Inhibitors of 15-lipoxygenase from orange peel. J. Agric. Food Chem..

[24] Barros L., Falcão S., Baptista P., Freire C., Vilas-Boas M., Ferreira I.C.F.R. (2008). Antioxidant activity of *Agaricus* sp. mushrooms by chemical, biochemical and electrochemical assays. Food Chem..

[25] Velhal S.G., Latpate R.V., Kulkarni S.D., Jaybhaye R.G. (2015). Taguchi design for parameter optimization of size-controlled synthesis of silver nanoparticles. Int. J. Emerg. Technol. Comput. Appl. Sci..

[26] Kim S.M., Park K.S., Kim K.D., Park S.D., Kim H.T. (2009). Optimization of parameters for the synthesis of bimodal Ag nanoparticles by Taguchi method. J. Ind. Eng. Chem..

[27] Kumari S.C., Selvakumar V., Padma P.N., Anuradha K. (2021). Optimization studies on green synthesis of silver nanoparticles from different plant extracts using Taguchi design. Indian J. Sci. Technol..

[28] Melkamu W.W., Bitew L.T. (2021). Green synthesis of silver nanoparticles using *Hagenia abyssinica* (Bruce) J.F. Gmel plant leaf extract and their antibacterial and anti-oxidant activities. Heliyon.

[29] Rakib-Uz-Zaman S.M., Hoque Apu E., Muntasir M.N., Mowna S.A., Khanom M.G., Jahan S.S., Akter N.R., Khan M.A., Shuborna N.S., Shams S.M. (2022). Biosynthesis of silver nanoparticles from *Cymbopogon citratus* leaf extract and evaluation of their antimicrobial properties. Challenges.

[30] Flieger J., Franus W., Panek R., Szymańska-Chargot M., Flieger W., Flieger M., Kołodziej P. (2021). Green synthesis of silver nanoparticles using natural extracts with proven antioxidant activity. Molecules.

[31] Nguyen V.P., Le Trung H., Nguyen T.H., Hoang D., Tran T.H. (2021). Synthesis of biogenic silver nanoparticles with eco-friendly processes using *Ganoderma lucidum* extract and evaluation of their theranostic applications. J. Nanomater..

[32] Singhal M., Chatterjee S., Kumar A., Syed A., Bahkali A.H., Gupta N., Nimesh S. (2021). Exploring the antibacterial and antibiofilm efficacy of silver nanoparticles biosynthesized using *Punica granatum* leaves. Molecules.

[33] Mat Yusuf S.N.A., Che Mood C.N.A., Ahmad N.H., Sandai D., Lee C.K., Lim V. (2020). Optimization of biogenic synthesis of silver nanoparticles from flavonoid-rich *Clinacanthus nutans* leaf and stem aqueous extracts. R. Soc. Open Sci..

[34] Verma S.K., Jha E., Panda P.K., Thirumurugan A., Suar M. (2019). Biological effects of green-synthesized metal nanoparticles: A mechanistic view of antibacterial activity and cytotoxicity. Advanced Nanostructured Materials for Environmental Remediation.

[35] Ssekatawa K., Byarugaba D.K., Kato C.D., Wampande E.M., Ejobi F., Nakavuma J.L., Maaza M., Sackey J., Nxumalo E., Kirabira J.B. (2021). Green strategy–based synthesis of silver nanoparticles for antibacterial applications. Front. Nanotechnol..

[36] Liu Y.-S., Chang Y.-C., Chen H.-H. (2018). Silver nanoparticle biosynthesis by using phenolic acids in rice husk extract as reducing agents and dispersants. J. Food Drug Anal..

[37] Aazam E.S., Zaheer Z. (2016). Growth of Ag-nanoparticles in an aqueous solution and their antimicrobial activities against Gram positive, Gram negative bacterial strains and *Candida* fungus. Bioprocess Biosyst. Eng..

[38] Franzolin M.R., Courrol D.d.S., de Souza Barreto S., Courrol L.C. (2022). *Eugenia uniflora* L. silver and gold nanoparticle synthesis, characterization, and evaluation of the photoreduction process in antimicrobial activities. Microorganisms.

[39] Kumar R.S., Kumar S.V., Lathiff M.K.M.A., Muthuboopathi G. (2019). Synthesis and characterization of bioinspired silver nanoparticles by aqueous leaf extract of *Indigofera cassioides*: Evaluation of antimicrobial and cytotoxic activity. J. Nanosci. Technol..

[40] Parlinska-Wojtan M., Kus-Liskiewicz M., Depciuch J., Sadik O. (2016). Green synthesis and antibacterial effects of aqueous colloidal solutions of silver nanoparticles using camomile terpenoids as a combined reducing and capping agent. Bioprocess Biosyst. Eng..

[41] Hayat J., Akodad M., Moumen A., Baghour M., Skalli A., Ezrari S., Belmalha S. (2020). Phytochemical screening, polyphenols, flavonoids and tannin content, antioxidant activities and FTIR characterization of *Marrubium vulgare* L. from 2 different localities of Northeast of Morocco. Heliyon.

[42] Erjaee H., Rajaian H., Nazifi S. (2017). Synthesis and characterization of novel silver nanoparticles using *Chamaemelum nobile* extract for antibacterial application. Adv. Nat. Sci. Nanosci. Nanotechnol..

[43] Kulikouskaya V., Hileuskaya K., Kraskouski A., Kozerozhets I., Stepanova E., Kuzminski I., You L., Agabekov V. (2022). Chitosan-capped silver nanoparticles: A comprehensive study of polymer molecular weight effect on the reaction kinetic, physicochemical properties, and synergetic antibacterial potential. SPE Polym..

[44] Balouiri M., Sadiki M., Ibnsouda S.K. (2016). Methods for in vitro evaluating antimicrobial activity: A review. J. Pharm. Anal..

[45] Kambale E.K., Nkanga C.I., Mutonkole B.-P.I., Bapolisi A.M., Tassa D.O., Liesse J.-M.I., Krause R.W.M., Memvanga P.B. (2020). Green synthesis of antimicrobial silver nanoparticles using aqueous leaf extracts from three Congolese plant species (*Brillantaisia patula*, *Crossopteryx febrifuga* and *Senna siamea*). Heliyon.

[46] Xing Y., Liao X., Liu X., Li W., Huang R., Tang J., Xu Q., Li X., Yu J. (2021). Characterization and antimicrobial activity of silver nanoparticles synthesized with the peel extract of mango. Materials.

[47] Khalil M.M.H., Ismail E.H., El-Baghdady K.Z., Mohamed D. (2014). Green synthesis of silver nanoparticles using olive leaf extract and its antibacterial activity. Arab. J. Chem..

[48] Difference between Gram Positive and Gram Negative Cell Wall. https://www.differencebetween.com/difference-between-gram-positive-and-gram-negative-cell-wall/.

[49] Hajipour M.J., Fromm K.M., Akbar Ashkarran A., Jimenez de Aberasturi D., Larramendi I.R.d., Rojo T., Serpooshan V., Parak W.J., Mahmoudi M. (2012). Antibacterial properties of nanoparticles. Trends Biotechnol..

[50] Morones J.R., Elechiguerra J.L., Camacho A., Holt K., Kouri J.B., Ramírez J.T., Yacaman M.J. (2005). The bactericidal effect of silver nanoparticles. Nanotechnology.

[51] Pal S., Tak Y.K., Song J.M. (2007). Does the antibacterial activity of silver nanoparticles depend on the shape of the nanoparticle? A study of the gram-negative bacterium *Escherichia coli*. Appl. Environ. Microbiol..

[52] Bruna T., Maldonado-Bravo F., Jara P., Caro N. (2021). Silver Nanoparticles and their antibacterial applications. Int. J. Mol. Sci..

[53] Dakal T.C., Kumar A., Majumdar R.S., Yadav V. (2016). Mechanistic basis of antimicrobial actions of silver nanoparticles. Front. Microbiol..

[54] Hu C., Ma S. (2018). Recent development of lipoxygenase inhibitors as anti-inflammatory agents. Medchemcomm.

[55] Kang K.-H., Liou H.-H., Hour M.-J., Liou H.-C., Fu W.-M. (2013). Protection of dopaminergic neurons by 5-lipoxygenase inhibitor. Neuropharmacology.

[56] Mashima R., Okuyama T. (2015). The role of lipoxygenases in pathophysiology; new insights and future perspectives. Redox Biol..

[57] Nuruki Y., Matsumoto H., Tsukada M., Tsukahara H., Takajo T., Tsuchida K., Anzai K. (2021). Method to improve azo-compound (AAPH)-induced hemolysis of erythrocytes for assessing antioxidant activity of lipophilic compounds. Chem. Pharm. Bull..

[58] Yoshioka Y., Li X., Zhang T., Mitani T., Yasuda M., Nanba F., Toda T., Yamashita Y., Ashida H. (2017). Black soybean seed coat polyphenols prevent AAPH-induced oxidative DNA-damage in HepG2 cells. J. Clin. Biochem. Nutr..

[59] Qin B., Yang K., Cao R. (2020). Synthesis, radical-scavenging qctivities, and protective effects against AAPH-induced oxidative damage in DNA and erythrocytes of piperine derivatives. J. Chem..

